# Social Transmission of Fear in Rats: The Role of 22-kHz Ultrasonic Distress Vocalization

**DOI:** 10.1371/journal.pone.0015077

**Published:** 2010-12-01

**Authors:** Eun Joo Kim, Earnest S. Kim, Ellen Covey, Jeansok J. Kim

**Affiliations:** 1 Department of Psychology, University of Washington, Seattle, Washington, United States of America; 2 Program in Neurobiology and Behavior, University of Washington, Seattle, Washington, United States of America; Université Pierre et Marie Curie, France

## Abstract

**Background:**

Social alarm calls alert animals to potential danger and thereby promote group survival. Adult laboratory rats in distress emit 22-kHz ultrasonic vocalization (USV) calls, but the question of whether these USV calls directly elicit defensive behavior in conspecifics is unresolved.

**Methodology/Principal Findings:**

The present study investigated, in pair-housed male rats, whether and how the conditioned fear-induced 22-kHz USVs emitted by the ‘sender’ animal affect the behavior of its partner, the ‘receiver’ animal, when both are placed together in a novel chamber. The sender rats’ conditioned fear responses evoked significant freezing (an overt evidence of fear) in receiver rats that had previously experienced an aversive event but not in naïve receiver rats. Permanent lesions and reversible inactivations of the medial geniculate nucleus (MGN) of the thalamus effectively blocked the receivers’ freeezing response to the senders' conditioned fear responses, and this occurred in absence of lesions/inactivations impeding the receiver animals' ability to freeze and emit 22-kHz USVs to the aversive event per se.

**Conclusions/Significance:**

These results—that prior experience of fear and intact auditory system are required for receiver rats to respond to their conspecifics' conditioned fear responses—indicate that the 22-kHz USV is the main factor for social transmission of fear and that learning plays a crucial role in the development of social signaling of danger by USVs.

## Introduction

Social signaling of imminent danger plays an adaptive (antipredatory) role in many species. For instance, ants use chemical signals to communicate threat to their colony [1]; chickens emit alarm calls that evoke escape responses in cohorts [2]; ground squirrels produce predator alarm calls to warn nearby conspecifics [3]; and monkeys vocalize predator-specific alarm calls that provoke threat-specific defensive behavior in group members [4]. It is generally believed that social distress signals increase group fitness and promote survival of the species [5].

Social signalling of danger (or fear) has also been investigated in laboratory rats (*Rattus norvegicus*). Since John W. Anderson's discovery in 1954 that adult rats emit a range of ultrasonic vocalizations (USV) [6], the 22-kHz USV has been found to be associated with various distress states [7], [8], [9], [10], [11], [12]. For instance, rats produce 22-kHz USV calls when subjected to stress and as a conditioned response (CR) following fear conditioning. Production of 22-kHz vocalizations is blocked by amygdalar lesions or inactivation [8], [13], [14], [15]. Chemical (e.g., carbachol) stimulation of the anterior hypothalamic-preoptic area also evokes 22-kHz vocalizations which are sonographically similar to USVs triggered via acoustic, tactile and footshock stimuli [16], [17]. Subsequent studies found that USV playback and artificial 22-kHz sine waves increase cell activities in the amygdala, the hypothalamus, the periaqueductal grey matter and the perirhinal cortex [18], [19], [20], brain structures implicated in defensive behavior.

Several studies have investigated whether 22-kHz USVs function as a social alarm call that can directly influence the behavior of conspecific rats. One of the earlier studies examined behaviors of a small group of rats living in seminatural visible burrow systems when confronted with a cat predator [21], [22]. In response to a cat, rats fled from the open space into the burrow system and emitted 22-kHz USVs that persisted ∼30 min after the cat was removed. However, when rats were individually confronted with the cat in visible burrow systems, they did not produce 22-kHz alarm calls, suggesting that 22-kHz USV production only emerges in the presence of familiar conspecifics or in a social setting (but see [23]). Other studies broadcasted the recorded 22-kHz USVs (in the absence of other stimuli) to individual rats and found conflicting results; one study reported that both onset and offset of USV playback decreased locomotor activity in conspecifics [24], but another study found that only the discontinuation of USV affected the conspecifics' behavior [16]. Thus, while these studies suggest that 22-kHz USV occurs under social situations, the specific role of 22-kHz USV as a social signal of danger remains unclear. Perhaps, the utilization of ultrasound playback to individual rats or the assessment of rats' behavior while they reside in separate burrows might not provide ideal conditions for detecting social transmission of fear in rats.

**Figure 1:**
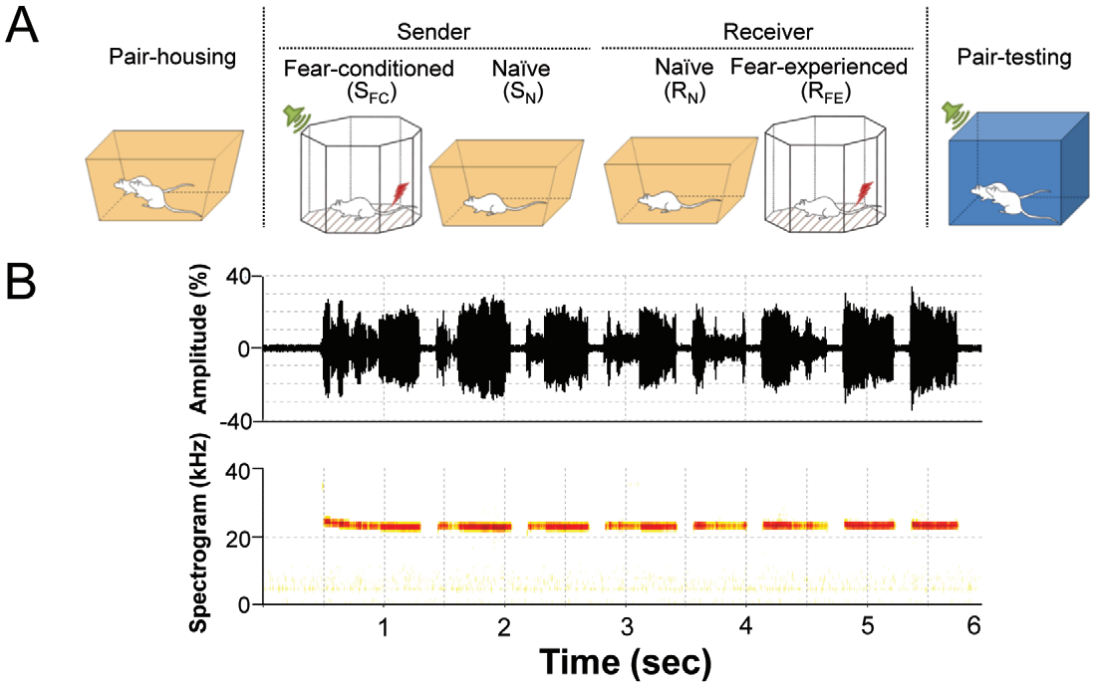
Social transmission of fear—experimental designs. (*A*) The sender (S) rats underwent 10 presentations of tone-footshock pairings (fear conditioned, S_FC_) while receiver (R) rats experienced either no footshock (naïve, R_N_) or 3 unsignaled footshocks (fear-experienced, R_FE_). Social transmission of fear was assessed by placing S and R rats together in a novel chamber and presenting the tone CS. (*B*) 22-kHz USV calls and spectrogram from a S_FC_ rat.

The present study employed pair-housed rats to directly test whether the conditioned fear-induced 22-kHz USVs emitted by the ‘sender’ rat produce fear behavior in its partner, the ‘receiver’ rat (Fig. 1). Specifically, we considered the possibility that the sender rat's USVs influence the receiver rat's behavior differently according to the partner animal's history of fear experience. Then, to verify whether 22-kHz USVs (and not other sensory factors) influence the partner's behavior, lesion and reversible inactivation techniques were applied on the auditory thalamus of the receiver rat to interrupt the primary auditory stream.

## Materials and Methods

### Ethics statement

All experiments were conducted in strict compliance to federal guidelines and as approved by the University of Washington Institutional Animal Care and Use Committee (animal assurance number A3464-01; protocol 4040-01, originally approved on 06.12.2008 – latest annual renewal approved 05.28.2010).

### Subjects

Experimentally naïve male Charles River Sprague-Dawley rats (initially weighing 275–300 gm) were pair-housed in our animal care facility (accredited by the Association for Assessment and Accreditation of Laboratory Animal Care) and maintained on a reverse 12-hr light-dark cycle (lights on at 19.00 hr) with free access to food and water. After 7 or 21 days of acclimation, animals were tested during the dark phase of the cycle.

### Medial geniculate nucleus (MGN) lesions and inactivation

Under anesthesia (30 mg/kg ketamine and 2.5 mg/kg xylazine, i.p.), rats were mounted in a stereotaxic instrument (Stoelting, Wood Dale, IL). Bilateral MGN lesions were made in receiver rats by passing constant current (1 mA, 15 sec; Ugo Basile, Comerio, Italy) through a stainless steel insect pin (#00) that was insulated with epoxy, except for ∼0.5 mm at the tip (coordinates from bregma: AP -5.3, ML ±3.4, DV -6.3 and AP -6.1, ML ±3.3, DV -6.5) [25], [26]. The partner sender rats received sham lesion surgery to match the postoperative recovery period (14 days). Guide cannulae (26 gauge; Plastics One, Roanoke, VA) were implanted bilaterally in the MGN (coordinates from bregma: AP -5.8, ML ±3.4, DV -5.6) of receiver-sender pair animals. Lidocaine (Sigma-Aldrich, St. Louis, MO), dissolved in artificial CSF (4%, pH ∼7.4) was microinfused into the MGN (bilaterally) by backloading the drug up a 33 gauge infusion cannula into polyethylene (PE 20) tubing connected to 10 µl Hamilton microsyringes (Hamilton Company, Reno, NV). The infusion cannulae protruded 1 mm beyond the guide cannula. An infusion volume of 1 µl (per side) was delivered using a Harvard PHD2000 syringe pump (Harvard Apparatus, South Natick, MA) over the course of 1 min (at a rate of 1 µl/min) [27], [28].

### Fear conditioning apparatus

Fear conditioning and pair-testing took place in two modular operant test chambers, each equipped with speaker modules and located in a sound-attenuating chest (Coulbourn Instruments, Allentown, PA). Fear conditioning chamber A was octagonal (26.5 cm diameter ×25 cm height) with all eight walls constructed of clear Plexiglas; the grid floor was composed of 17 stainless steel bars (5 mm diameter) spaced 15 mm center-to center, wired to a Coulbourn precision-regulated animal shocker and wiped with 5% ammonium hydroxide solution at the beginning of each trial. Pair-testing chamber B was rectangular (27 cm width ×28 cm length ×30.5 cm height) with front and back walls made of clear Plexiglas and two side walls made of metal plates; the floor was made of smooth Plexiglas and wiped with 1% acetic acid solution at the beginning of each trial.

### Behavioral procedures

Animals were divided into one of the following five paired groups: (i) Sender-fear conditioned and Receiver-naïve, S_FC_-R_N_; (ii) Sender-fear conditioned and Receiver-fear experienced, S_FC_-R_FE_; (iii) Sender-naïve and Receiver-fear experienced, S_N_-R_FE_; (iv) Sender-fear conditioned and Receiver mgn lesioned-fear experienced, S_FC_-R_MGN(LS)-FE_; and (v) Sender-fear conditioned and Receiver mgn lidocaine-fear experienced, S_FC_-R_MGN(LI)-FE_.

#### S_FC_-R_N_

On day 1, S_FC_ animals were placed in fear conditioning chamber A. After 2 min, they were presented with 10 tone conditioned stimuli (CS: 2.9 kHz, 82 dB, 20 sec) that began 19 sec before the footshock unconditioned stimulus (US: 2 mA, 1 sec), and terminated at the same time as the US. CS-US pairings were presented with 2 minute inter-trial intervals (ITIs). Animals were removed 2 min after the last shock and returned to their home cages. This fear conditioning procedure produced robust postshock USV and conditioned USV to the tone CS in all animals. While S_FC_ rats were undergoing tone fear conditioning, the partner R_N_ rats remained in their home cages. On day 2, the paired S_FC_ and R_N_ animals were placed together in testing chamber B, where 1 min of baseline was followed by 8 minutes of continuous tone CS.

#### S_FC_-R_FE_

On day 1, S_FC_ rats underwent tone fear conditioning as described above. The partner R_FE_ rats were placed in chamber A where after 3 min, 3 unsignaled footshocks (1 mA, 1 sec, 60 sec apart) were presented to produce a moderate fear experience [29]. One minute after the last shock, R_FE_ rats were returned to their home cages. On day 2, the paired S_FC_ and R_FE_ animals were tested in chamber B.

#### S_N_-R_FE_

On day 1, R_FE_ animals experienced 3 unsignaled footshocks in chamber A (described above), while S_N_ animals remained in their home cages. On day 2, S_N_-R_FE_ rat pairs were tested in chamber B.

#### S_FC_-R_MGN(LS)-FE_

These animal pairs underwent the same fear conditioning (day 1) and pair-testing (day 2) as the S_FC_-R_FE_ animals.

#### S_FC_-R_MGN(LI)-FE_

On day 1 (in chamber A), S_FC_ rats underwent tone fear conditioning as previously described (no drug infusions), while R_MGN(LI)-FE_ animals received lidocaine infusions into their MGN just prior to experiencing 3 unsignaled footshocks. On day 2, the paired S_FC_-R_MGN(LI)-FE_ rat pairs were tested in chamber B (no drug infusions). On day 3, S_FC_ animals received lidocaine infusions into their MGN prior to testing alone in chamber B. On day 4, S_FC_ animals were retested in chamber B without drug infusions.

### Histology

At the completion of behavioral testing, the R_MGN(LS)-FE_ and R_MGN(LI)-FE_ animals were overdosed with a ketamine-xylazine cocktail and perfused intracardially with 0.9% saline followed by 10% buffered formalin. The brains were removed and stored in 10% formalin overnight and then kept in 30% sucrose solution until they sank. Transverse sections (50 µm) were taken through the extent of the lesion, mounted on gelatin-coated slides, and stained with cresyl violet and Prussian blue dyes.

### Freezing and USV data collection and analyses

The CS and US presentations were controlled by a PC equipped with the Coulbourn LabLinc Habitest Universal Linc System. The automated collection of USV and freezing data has been previously described in detail [15]. Each session was also recorded for video and audio off-line analysis (for pair-testing) using an infrared light source and miniature video camera (CB-21; Circuit Specialists, Inc., Mesa, AZ).

During fear conditioning, a 24 cell infrared activity monitor (mounted on top of the chamber) that detects the movement of the emitted infrared (1300 nm) body-heat image from the animals in the *x*-, *y*-, and *z*-axes was used to assess freezing behavior. In brief, the total time of inactivity exhibited by each animal was measured using a computer program, and freezing was defined as continuous inactivity lasting ≥3 sec. Any behavior that yielded an inactivity period of <3 sec was recorded as general activity. During pair-testing, a custom-written computer-assisted scoring program (in C language) was used by an uninformed observer to score the duration of freezing displayed by each animal by manual keystrokes on the computer keyboard. The sender and receiver rats were distinguished by Sharpie markings on their fur. Freezing was defined as the absence of any visible movement of the body and vibrissae except for movement necessitated by respiration [30], and filtered by the C program with 3-sec threshold as used in the infrared activity analysis. The percent freezing was computed as ((total duration of freezing
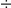
total duration of observation) ×100.

A heterodyne bat detector (Mini-3; Noldus Information Technology, Wageninge, The Netherlands) was used to transform high-frequency (22±5 kHz) ultrasonic vocalizations into the audible range. The output of the bat detector was fed through an audio amplitude filter (Noldus), which filtered out signals falling below an amplitude range that was individually adjusted for each animal. The resulting signal was then sent to an IBM PC equipped with Noldus UltraVox vocalization analysis software. The software converted the signal into vocalization onset and offset times according to the following specifications: an onset was recorded if the duration of sound was ≥30 msec, and an offset was recorded if the onset of the ensuing episode was ≥40 msec later. If the interval was <40 msec, the two bouts were counted as a single episode. The percent USV was computed as ((total duration of USV
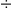
total duration of observation) ×100. Note that during the S-R pair-testing, the total USV detected technically cannot be separated into sounds produced by sender vs. receiver rats. D980 Ultrasound Detector and BatSound Pro (version 3.3) real-time spectrogram software (Pettersson Elektronik AB, Uppsala, Sweden) were used to show sample conditioned fear-induced USV calls.

### Statistical analyses

The freezing and USV data collected during 10 tone-footshock fear conditioning (day 1), 3 unsignaled footshock fear experiences (day 1) and the sender-receiver pair-testing (day 2) were analyzed by ANOVA followed by Bonferroni post hoc test. The pair-testing data were further analyzed from the first 3 min of the tone when the fear responses were maximal. Data from 1- and 3-week pair-housed groups were compared by Independent *t*-test. The comparison between S_FC_ rats' drug conditions (with and without lidocaine injection) was conducted by one-sample *t*-test. Pearson's correlation coefficient was calculated to assess the relationship between the fear-experienced receiver rats' USV onset latency during 3 unsignaled footshocks and their percent freezing during pair-testing.

## Results

### Effects of sender rats' fear responses on naïve receiver rats

**Figure 2:**
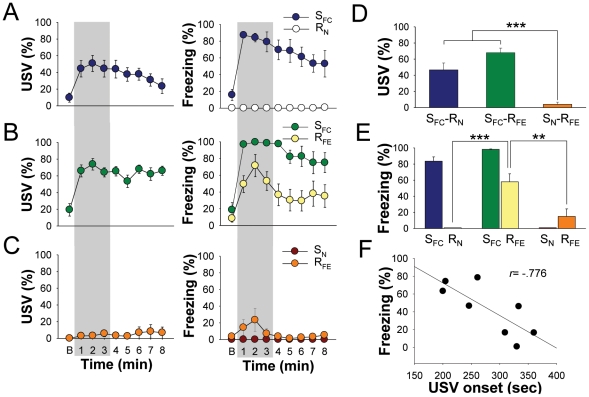
Results from three groups of sender-receiver pairs (n = 16 rats/pair). fear-conditioned sender and naïve receiver (S_FC_-R_N_); fear-conditioned sender and fear-experienced receiver (S_FC_-R_FE_); and naïve sender and fear-experienced receiver (S_N_-R_FE_). (*A–C*) Mean (± SEM) percentage time spent emitting USV and displaying freezing by S and R rats during baseline and 8 min tone CS presentation. (*D, E*) Group differences in USV (F_2,23_ = 28.583, *P*<.0001) and freezing (F_5,47_ = 52.743, *P*<.0001) during the first 3-min period of the pair-testing (grey sections, *A* through *C*). *, ** and *** denote *P*<.05, *P*<.001 and *P*<.0001, respectively, Bonferroni test. (*F*) A significant correlation between R_FE_ animals' USV onset latencies during 3 unsignaled footshocks on day 1 and the R_FE_ animals' freezing levels during S_FC_-R_FE_ pair-testing on day 2 (*r* = −.779, *P* = .02).

Sender rats (S_FC_) robustly acquired conditioned fear, as assessed by freezing and 22-kHz USV, when presented with 10 pairings of a tone conditioned stimulus (CS: 2.9 kHz, 82 dB, 20 sec) that coterminated with a footshock unconditioned stimulus (US: 2 mA, 1 sec) (Fig. S1 *A* and *B*). A day later, when the sender rat and its naïve receiver (R_N_) partner were placed together in a novel chamber, both initially exhibited exploratory behavior. However, when the tone CS came on, the S_FC_ rats immediately displayed freezing and USV that persisted through the entire 8 min of testing (Fig. 2*A*). During this time, the R_N_ rats continued their exploratory behavior and exhibited no sign of freezing. This was true whether the animals were pair-housed for 1 or 3 weeks; the sender rats' robust conditioned fear responses of freezing and USV had virtually no effects on the naive receiver rats' ongoing behavior (Fig. S2).

### Effects of sender rats' fear responses on fear-experienced receiver rats

The sender rats (S_FC_) were trained in the same manner as described above, whereas the receiver rats underwent an episode of fear experience (R_FE_) consisting of 3 unsignaled footshocks (1 mA, 1 sec, 60 sec apart) in the absence of any other rats (Fig. S1 *C* and *D*). When the S_FC_ and the R_FE_ rats were placed together in the novel chamber the next day, both displayed exploratory behavior. When the tone CS came on, the S_FC_ again promptly froze and emitted USV (Fig. 2*B*). In response to the sender rats' fear behavior, the R_FE_ rats also exhibited freezing, which was virtually absent in R_N_ rats. The total amount of USV in this pair was high throughout the session because the freezing receiver rats also emitted USVs; the bat detector cannot discriminate USVs from S_FC_ and R_FE_ animals and therefore accumulated collectively. However, at least during the first 3-min period of the tone presentation, the USV levels were comparable between S_FC_-R_N_ and S_FC_-R_FE_ pairs (Fig. 2*D*).

It is unclear, however, whether the freezing displayed by the R_FE_ rats was a result of the S_FC_ rats' fear responses or a sensitized reaction to the novel tone stimulus. The latter possibility is unlikely because R_FE_ rats did not freeze in response to the tone stimulus if the sender rats did not demonstrate fear responses (S_N_; Fig. 2*C*). The fact that R_FE_ rats rarely froze during the whole pair-testing session—the initial exploration and the tone presentations in the novel chamber—also excludes the possibility of generalized anxiety (due to the unsignaled shock experience on the previous day) influencing the R_FE_ rats' behavior. Thus, during the S-R pair-testing, the sender rats' CRs to the tone CS (and not the tone stimulus per se) caused freezing in fear-experienced receiver rats (first 3-min data: Fig. 2*D and E*).

Additionally, the amount of freezing displayed by the R_FE_ rats during the entire S-R pair-testing session was positively correlated with the magnitude of USV the R_FE_ rats emitted during the episode of fear experience (Fig. 2*F*). Specifically, their USV onset latencies on day 1 fear experience were significantly correlated with the freezing levels on day 2 pair-testing (*r* = −.779, *P* = .02).

### Effects of sender rats' fear responses on receiver rats with MGN lesions

**Figure 3:**
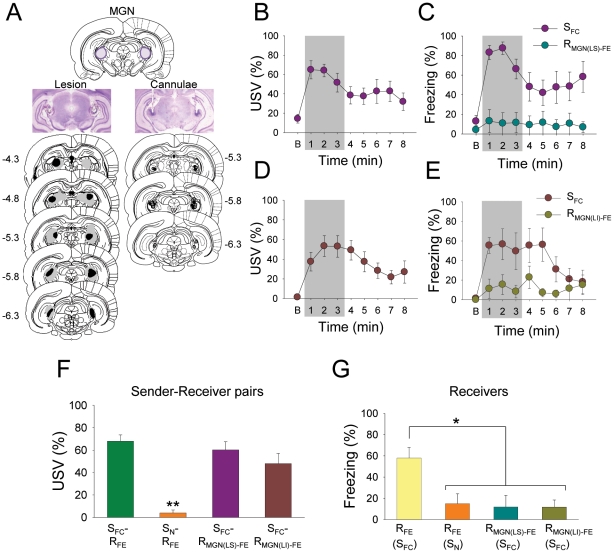
MGN lesion, MGN inactivation and social transmission of fear. (*A*) Photomicrograph and histological reconstruction of MGN lesions (solid and gray represent minimum and maximum extent of the lesions, respectively) and cannulae implantation (each dot represents the injection site in the MGN of the S_FC_ (grey) - R_MGN(LI)-FE_ (black) pair). (*B, C*) Mean percentage USV and freezing from S_FC_ (n = 8) and MGN-lesioned receiver (R_MGN(LS)-FE_, n = 8) rats during the pair-testing. (*D, E*) Mean percentage USV and freezing from S_FC_ (n = 7) and MGN-lidocaine receiver (R_MGN(LI)-FE_, n = 7) rats during the pair-testing. (*F, G*) Group differences in USV between S_FC_-R_FE_, S_N_-R_FE_, S_FC_-R_MGN(LS)-FE_, and S_FC_-R_MGN(LI)-FE_ pairs (*F*_3,30_ = 24.681, *P*<.0001) and freezing between fear-experienced receiver groups (*F*_3,30_ = 5.770, *P*<.01) during the first 3-min period of the pair-testing (grey sections, *B* through *E*). * denotes *P*<.05 (Bonferroni test); ** denotes *P*<.001 compared to the other three pairs (Bonferroni test).

To definitively identify the crucial feature of S_FC_ rats' behavior that produces freezing in fear-experienced receiver rats, the primary auditory pathway was disrupted in receiver rats via bilateral lesions of the MGN of the thalamus (Fig. 3*A*). The MGN lesions selectively prevent auditory information flow to the forebrain, while leaving the brainstem auditory reflex pathways intact, and without altering other sensory information systems. The MGN-lesioned receiver (R_MGN(LS)-FE_) rats demonstrated postshock freezing to 3 unsignaled footshocks which was comparable to intact rats (i.e., R_FE_ rats paired with S_FC_ rats, R_FE_ rats paired with S_N_ rats) (Text S1). When the S_FC_ and the R_MGN(LS)-FE_ rats were placed in a novel chamber, both displayed exploratory behavior during the baseline period. As before, the tone CS promptly evoked freezing and USV in the S_FC_ rats (Fig. 3 *B* and *C*). However, unlike the intact R_FE_ rats, the R_MGN(LS)-FE_ rats failed to freeze in the presence of the sender rats' fear responses. Although the R_MGN(LS)-FE_-S_FC_ pairs and the intact R_FE_-S_FC_ pairs produced similar levels of USV (Fig. 3*F*), the freezing behavior was virtually absent in the R_MGN(LS)-FE_ animals (Fig. 3*G, P*<.05). Because the R_MGN(LS)-FE_ rat's forebrain could not process USV due to the MGN lesions, this indicates that the critical factor that evokes fear response in the receiver rat was not the visible freezing or the odor produced by the sender rat, but rather the sender's USV.

### USV effects on receiver rats with MGN inactivation during fear experience

To understand the significance of a positive correlation between the onset latency of USVs emitted by the receiver rats during an episode of fear experience on Day 1 and their freezing levels during the pair-testing on Day 2 (Fig. 2*F*), sender and receiver rats were implanted with guide cannulae aimed at the MGN bilaterally (Fig. 3*A*). The sender (S_FC_) rats were trained in the same manner as before (Fig. S1 *A* and *B*), whereas the receiver rats (R_MGN(LI)-FE_) received intra-MGN infusions of lidocaine just prior to undergoing an episode of fear experience. During the 3 unsignaled footshocks, intra-MGN lidocaine affected neither postshock freezing nor USV responses of the R_MGN(LI)-FE_ rats (Fig. S1 *C* and *D*). During the pair-testing, both the S_FC_ and the R_MGN(LI)-FE_ rats (sans lidocaine) initially displayed exploratory behavior in the novel chamber. When the tone CS came on, the S_FC_ rats exhibited conditioned freezing and USV responses (Fig. 3*D and E*). However, the R_MGN(LI)-FE_ rats displayed significantly impaired freezing in response to the sender rats' fear behavior (Fig. 3*G, P*<.05). Thus, the receiver rats' ability to process their own USVs (emitted during the 3 unsignaled footshocks) somehow seems to be crucial for responding to their conspecifics' USVs in a different context.

>The intra-MGN lidocaine temporarily interfered with auditory information flow to the forebrain because the S_FC_ rats displayed significantly attenuated freezing and no USVs to the tone CS when their MGNs were inactivated with lidocaine (Text S1 and Fig. S3 *A* and *B*). Once the MGN returned to the normal state (next day), the S_FC_ rats displayed considerable freezing and USVs to the CS during re-testing, though which may have resulted in extinction.

## Discussion

Our findings indicate that classically conditioned fear-induced 22-kHz USV calls emitted by sender rats convey fear-eliciting information to receiver rats that previously experienced an aversive event, but not to naïve receiver rats. Specifically, fear experienced rats displayed freezing (an index of fear) in response to their partner's USVs. Because the rats were pair-housed, the experimental design minimized extraneous factors such as novelty and aggression associated with testing unfamiliar rats together. In naïve S-R pairs, despite the fact that the sender rat displayed robust freezing and USV to the tone CS, the naïve receiver rat's ongoing behavior in the novel chamber continued unaltered. Conversely, the naïve receiver rat's exploratory behavior had no effect on the sender's freezing and USV, even when the receiver rat stepped over and attempted to burrow under the sender rat. It appears then that, though rats are predisposed to respond to USV [31], some prior knowledge of fear experience on the part of the receiver rat is required for the sender rat's fear response to effectively trigger fear in the receiver rat. Consistent with this view, a recent study also found that previous experience of avoidance learning is required for the social transmission of avoidance behavior in rats [32].

The freezing (fear) response displayed by the previously fear-experienced receiver rats during the pair-testing was produced by the sender rats' fear responses to the tone stimulus. This conclusion is supported by control experiments, which indicate that the fear-experienced receiver rats did not freeze to the novel tone (presented in a novel chamber) if the sender rats did not demonstrate fear responses, hence excluding the possibilities of a sensitized reaction and carry-over generalized anxiety (from the previous day's unsignaled footshock experience) to novelty stimuli.

The observation that MGN-lesioned receiver rats showed no fear response in the company of sender rats that displayed robust freezing and 22-kHz USV indicates that the receiver rats are responding directly to the sender rats' USV calls. With no MGN to process USVs, the sender rats' freezing and other fear responses, such as odor (but see [33] and [34]), do not influence the receiver rats. Hence, the 22-kHz USV is a crucial feature of social transmission of fear in rats. The fear-evoking properties of USV in rats appear to require processing in the primary auditory system at or above the level of the thalamus, although the possible contribution of the MGN projections to the auditory brain stem cannot be ruled out. A recent study has shown that fear conditioning to a 22-kHz USV CS in rats requires higher cortical (i.e., the perirhinal cortex) projections to the amygdala [35], unlike fear conditioning to a pure tone CS which can be fully supported by either cortical projections to the amygdala or direct projections from the medial division of MGN to the amgydala [25], [26], [36]. The finding that USV also induces c-fos expression in auditory and perirhinal cortices [20] further suggests that rats' responses to USV [31] are mediated upstream from the MGN.

**Figure 4:**
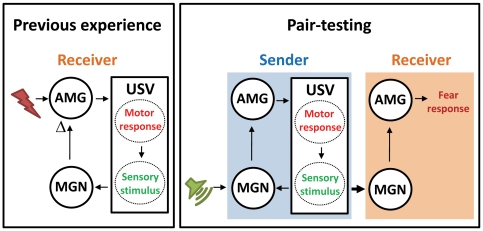
A hypothesized model of auto-conditioning. (*Left*) The ‘receiver’ rat reflexively emits USV response during unsignaled footshock experience. The USV serves as an internally-generated CS that acquires associative strength (Δ) in the amygdala (AMG) by virtue of pairings with succeeding footshock US. (*Right*) During pair-testing, the USV generated by the ‘sender’ animal will function as an effective CS to the receiver animal, producing a conditioned fear response.

What is the basis, then, for the sender's USV affecting the behavior of fear-experienced, but not naïve, receiver rats? In fear-experienced receivers, the USVs that they emitted during 3 unsignaled footshocks seem to prime them to experience fear during the sender-receiver pair-testing the next day (Fig. 2*F*). Specifically, their USV onset latencies during fear experience on day 1 were significantly correlated with the freezing levels on day 2 pair-testing. This suggests an intriguing possibility that the receiver rat's reflexively emitted USVs (to unsignaled footshocks) served as an internally-generated CS that formed ‘auto-conditioning’ with the subject's fear state (Fig. 4). If that were the case, then the receiver rats that emitted USVs early on, starting with the first footshock, would acquire a stronger fear association than those that emitted USVs after the second or third footshock (Fig. S4 *A*), based on the traditional view of CS-US contingency in the classical conditioning [37]. Indeed, during the pair-testing, the receiver rats that started to emit USVs prior to the second or last shock on day 1 froze significantly more than those that started to emit USVs after the last shock (Fig. S4 *B*). The USV-footshock auto-conditioning is likely to occur in the amygdala, a structure implicated in acquisition and expression of conditioned fear response [13], [15], or via auditory thalamus which has been also considered as a site of plasticity for auditory fear conditioning [38], [39]. Later, during the pair-testing, the sender's conditioned USV response (emitted to the tone CS) would serve as an effective surrogate CS [35] to evoke the representation of previous USV-fear association in the receiver animal, via the MGN-mediated amygdalar activation. In support of this notion is the finding that temporarily inactivating the MGN of receiver rats (via targeted infusions of lidocaine, a voltage-gated Na^+^ channel blocker) during 3 unsignaled footshocks, did not affect their ability to display postshock freezing and USV but did prevent the sender's USV from affecting their behavior in the paired experiments. Presumably this was because lidocaine prevented the transmission of USV information at or above the level of the thalamus, so that the USV-fear association was not formed. Consequently, even with fully functional MGNs during the pair-testing, the receiver rats did not freeze to the sender rats' USV emission. Obviously, manipulations that can selectively inhibit USVs without altering other postshock fear responses (currently unavailable) will be required to further test this notion of auto-conditioning, perhaps analogous to auditory mirroring hypothesized in birds [40].

As mentioned above, there was no evidence of social transmission of fear when naïve rats were tested with fear conditioned partner rats; the naïve rats did not freeze when placed in a novel chamber and presented with a novel tone nor did they freeze when the partner rats displayed robust freezing and USV responses. However, a recent study reported that 10 minutes of social interaction with recently fear conditioned rats in the home cage enhanced the naïve partner rats' subsequent fear conditioning, suggesting that social transfer or modulation of fear transpired during brief social interaction [41]; see also [42]. In our study, a relatively prolonged ∼3 hours of interaction with recently fear conditioned sender rats in the homecage did not enhance the receiver rats' conditioned freezing to the 3 unsignaled footshocks when compared to receivers that interacted with naïve senders (Fig. S1 *C*). The social transfer of fear was evident only when USV calls were emitted by the partner sender rats during the paired testing. Whether brief versus prolonged social interaction between recently fear conditioned rats and partner rats about to undergo fear conditioning is a pivotal factor in social transfer of fear requires further investigation.

In summary, the present findings provide direct evidence that conditioned fear-induced 22 kHz USV emitted by one rat evokes fear behavior in a partner rat, provided that the receiver rat had previously experienced fear. Hence, experience-dependent fear responses to the USV in pair-housed laboratory rats might serve as a useful animal model towards understanding the neurobiological basis of normal and aberrant social fear learning in humans [43].

## Supporting Information

Figure S1 Mean percentage (± SEM) freezing (*A*) and USV (*B*) displayed by S_FC_ rats during auditory fear conditioning (10 tone-footshock pairings), and mean percentage freezing (*C*) and USV (*D*) by R_FE_ rats during fear experience (3 unsignaled footshocks.
(TIF)

Figure S2 Mean percentage (± SEM) freezing during pair-testing by S_FC_ and R_N_ rats that were pair-housed for either 1 week or 3 weeks prior to the experiment. There was no effect of the duration of pair-housing.
(TIF)

Figure S3 Mean percentage (± SEM) freezing (*A*) and USV (*B*) displayed during first 3-min period of the tone testing on day 3 by S_FC_ animals injected with lidocaine in their MGNs (S_FC-LI_) and when re-tested in drug-free state (S_FC-no drug_) on day 4.
(TIF)

Figure S4 The time course of USV emitted by individual receiver rats during three unsignaled shocks (represented by filled triangles) and their subsequent freezing level during the pair-testing (*A*). Eight rats were divided into USV-footshock ‘paired’ and USV-footshock ‘unpaired’ groups depending on whether their USV preceded and overlapped with footshock(s). (*B*) Mean percentage (± SEM) freezing displayed during pair-testing by ‘paired’ and ‘unpaired’ R_FE_ groups.
(TIF)

Text S1 Supporting Text.
(DOC)
