# Supplementary figures and images for: Social Transmission of Fear in Rats: The Role of 22-kHz Ultrasonic Distress Vocalization
Source: PLoS One. 2010 Dec 1;5(12):e15077. doi: 10.1371/journal.pone.0015077 (PMC2995742; doi:10.1371/journal.pone.0015077)

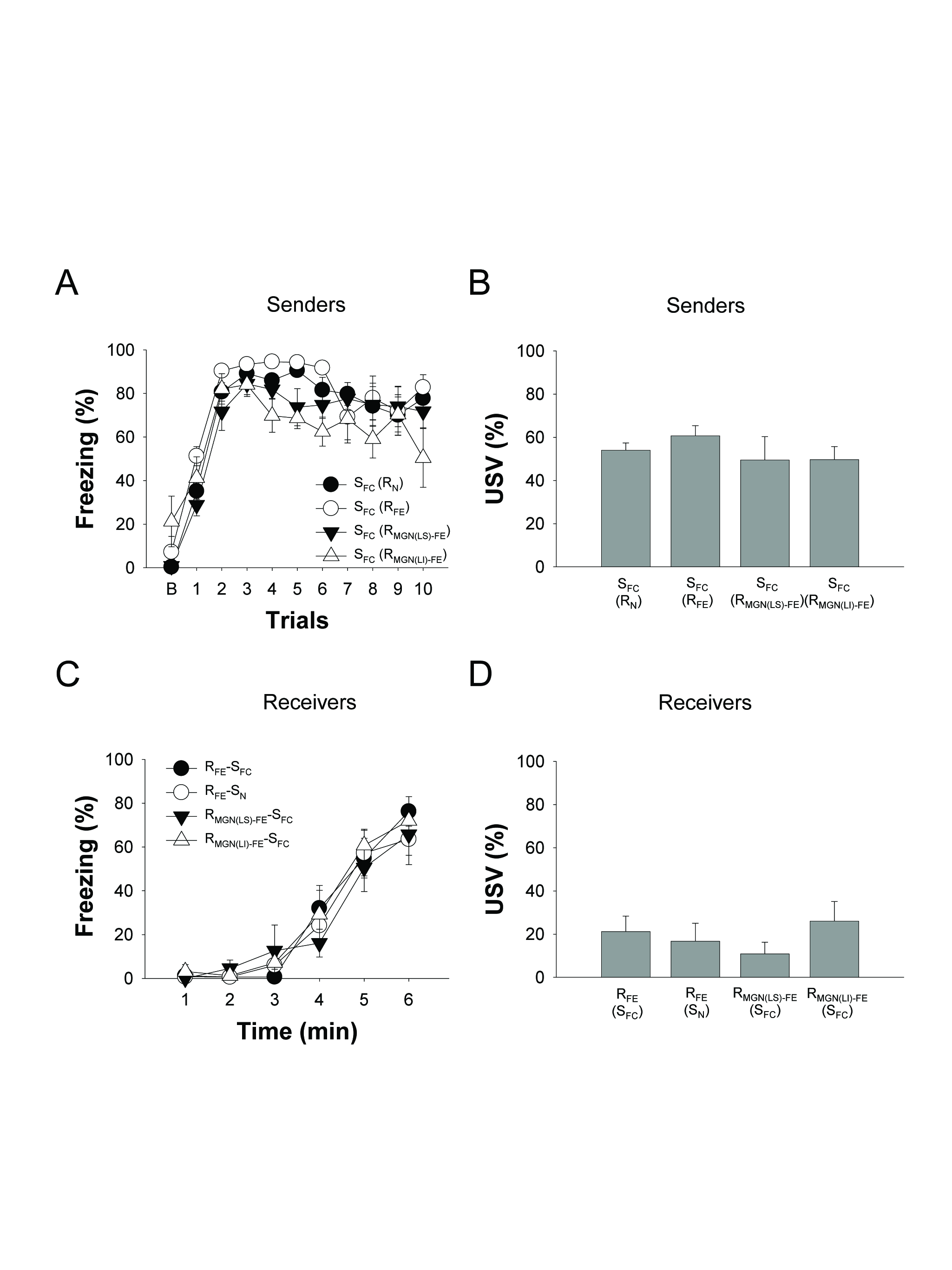

Supplement: Figure S1 — Mean percentage (± SEM) freezing (A) and USV (B) displayed by SFC rats during auditory fear conditioning (10 tone-footshock pairings), and mean percentage freezing (C) and USV (D) by RFE rats during fear experience (3 unsignaled footshocks. (TIF) [file pone.0015077.s001.tif]

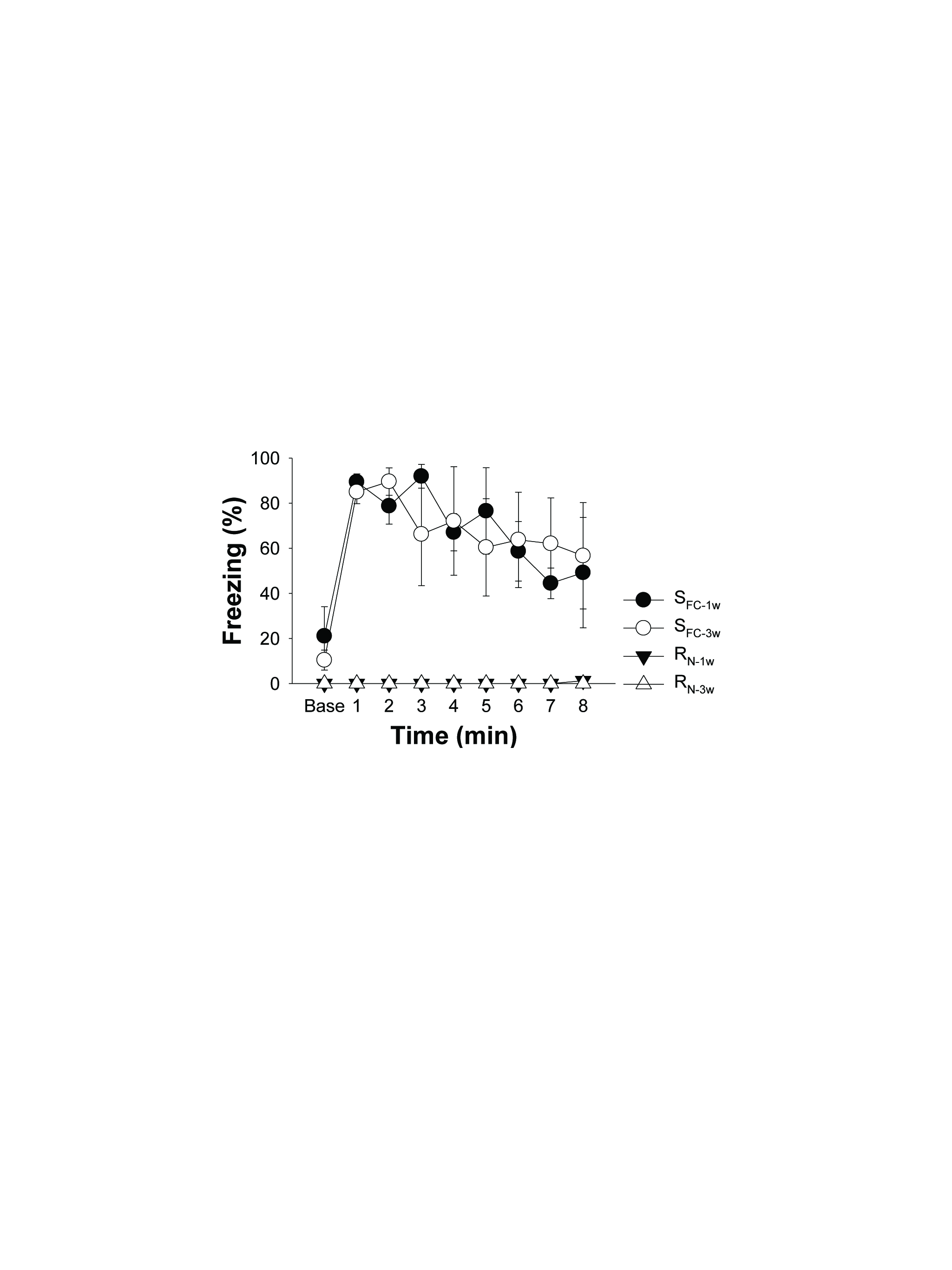

Supplement: Figure S2 — Mean percentage (± SEM) freezing during pair-testing by SFC and RN rats that were pair-housed for either 1 week or 3 weeks prior to the experiment. There was no effect of the duration of pair-housing. (TIF) [file pone.0015077.s002.tif]

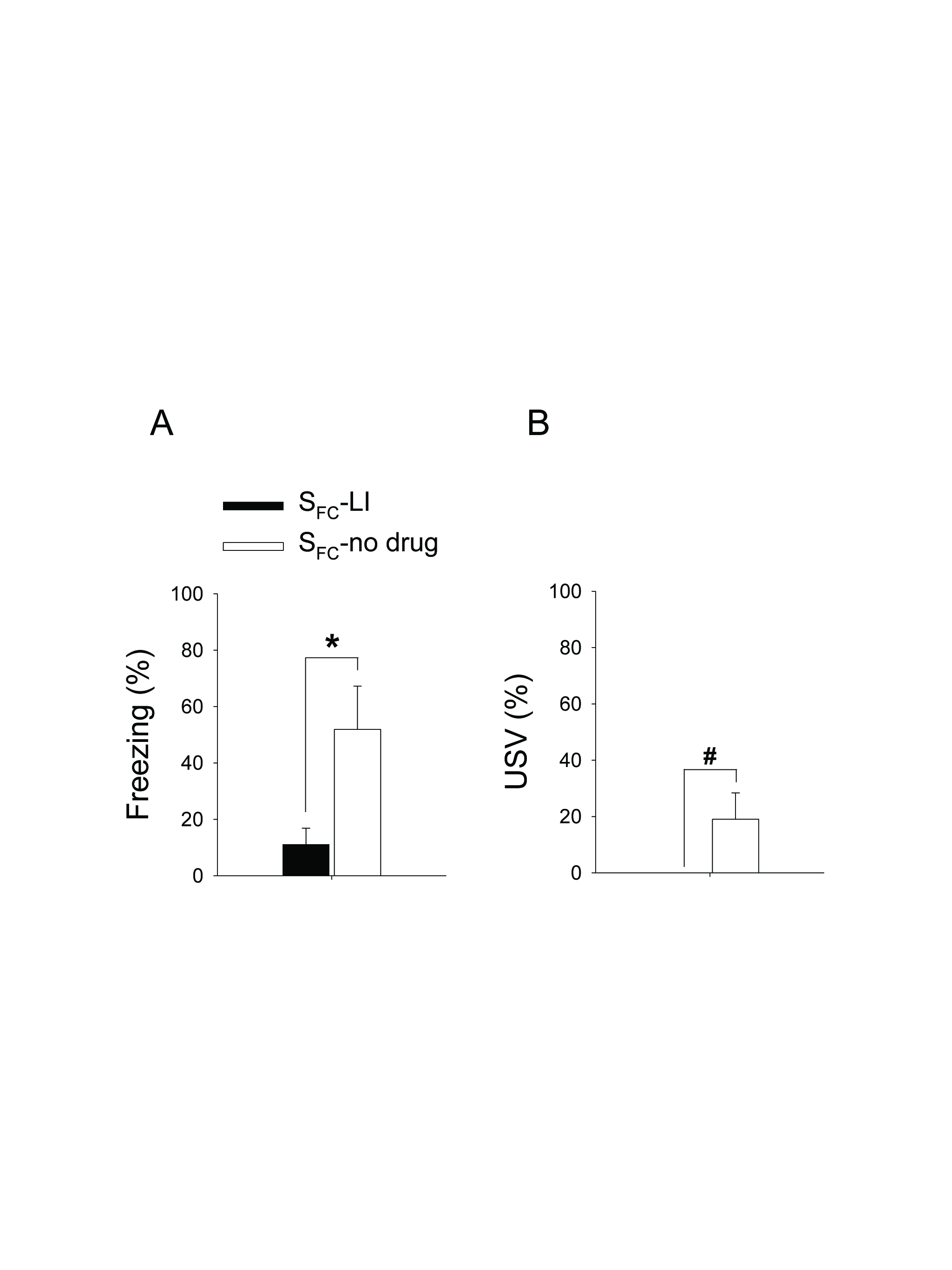

Supplement: Figure S3 — Mean percentage (± SEM) freezing (A) and USV (B) displayed during first 3-min period of the tone testing on day 3 by SFC animals injected with lidocaine in their MGNs (SFC-LI) and when re-tested in drug-free state (SFC-no drug) on day 4. (TIF) [file pone.0015077.s003.tif]

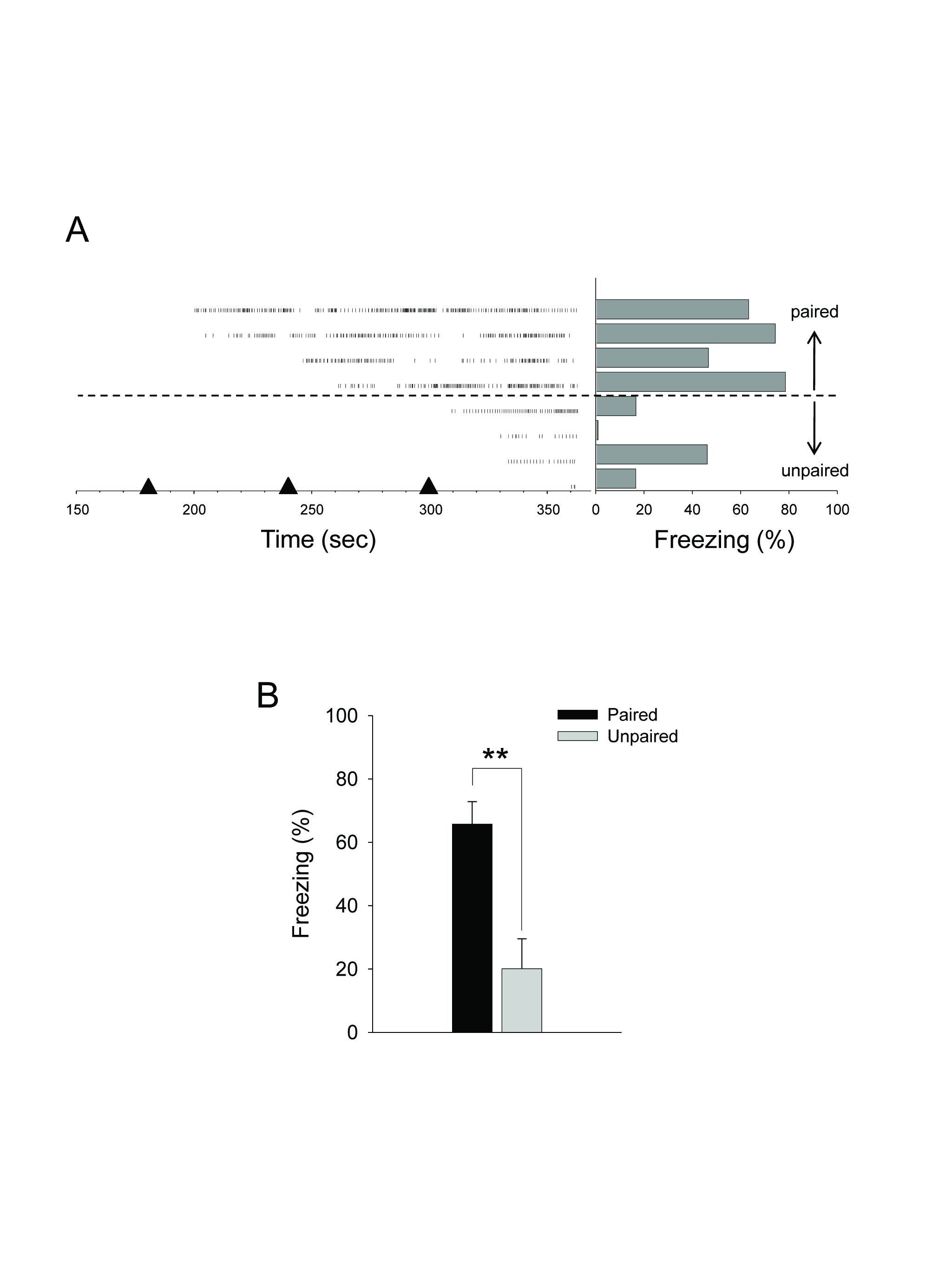

Supplement: Figure S4 — The time course of USV emitted by individual receiver rats during three unsignaled shocks (represented by filled triangles) and their subsequent freezing level during the pair-testing (A). Eight rats were divided into USV-footshock ‘paired’ and USV-footshock ‘unpaired’ groups depending on whether their USV preceded and overlapped with footshock(s). (B) Mean percentage (± SEM) freezing displayed during pair-testing by ‘paired’ and ‘unpaired’ RFE groups. (TIF) [file pone.0015077.s004.tif]
