# Supplementary material for: Social Transmission of Fear in Rats: The Role of 22-kHz Ultrasonic Distress Vocalization
Source: PLoS One. 2010 Dec 1;5(12):e15077. doi: 10.1371/journal.pone.0015077 (PMC2995742; doi:10.1371/journal.pone.0015077)
Supplement: Text S1 — Supporting Text. (DOC) [file pone.0015077.s005.doc]

**Supporting text**

**Text S1**

Supporting figure 1*A* shows the mean percentage freezing (SEM) displayed by S_FC_ rats that were partnered with R_N_, R_FE_ , R_MGN(LS)-FE_, or R_MGN(LI)-FE_ rats during tone fear conditioning on day 1. There were no reliable differences among S_FC_ groups during the baseline (*F*_3, 30_ = 2.156, *P* = .116) and 10 CS and ITI periods (*F*_3, 30_ = 2.541, *Ps* > .05).

Supporting figure 1*B* shows the mean percentage USV displayed by S_FC_ rats during tone fear conditioning on day 1. There were no significant differences among the four sender groups during 10 CS and ITI periods (*F*_3, 30_ = .587, *P* = .629).

Supporting figure 1*C* presents the mean percentage freezing displayed by R_FE_ (paired with either S_FC_ or S_N_), R_MGN(LS)-FE_ (paired with S_FC_), and R_MGN(LI)-FE_ (paired with S_FC_) rats during 3 unsignaled footshocks on day 1. No group differences were observed during the 3 min baseline (*F*_3, 30_ = .569, *P* = .641) and 3 ITI periods (*F*_3, 30_ = .430, *P* = .733).

Supporting figure 1*D* shows the mean percentage USV displayed by R_FE_ groups as in Fig. S1*C*. The levels of USV during 3 ITI periods were comparable in the four receiver groups (*F*_3, 30_ = .715, *P* = .552).

Supporting figure 2 displays the mean percentage freezing levels of S_FC_ and R_N_ animals that were pair-housed for either 1 or 3 weeks prior to the experiment. The duration of pair-housing had no effect on freezing for both S_FC_ and R_N_ rats during pair-testing on day 2 (both *P’*s *>* .3, by independent *t*-test).

Supporting figure 3 represents the mean percentage freezing (Fig. S3*A*) and USV (Fig. S3*B*) displayed by S_FC_ rats with intra-MGN lidocaine (on day 3) and without the drug (on day 4) during the first 3-min period of the tone testing. Despite re-testing, which may result in extinction, the S_FC_ rats froze more (*P* < .05, by one sample *t*-test) and tended to emit USVs more (*P* = .089, by one sample *t*-test) when they were in drug-free state (on day 4) than when they were injected with lidocain in their MGN (on day 3).

Supporting figure 4*A* shows individual (R_FE_) rat’s time course of USV emission during the unsignaled shock episode and their subsequent level of freezing during the pair-testing (S_FC_-R_FE_). Four R_FE_ rats received footshocks while emitting USVs (‘paired’ USV-footshock experience), while the other four rats received footshocks prior to emitting USVs (‘unpaired’ USV-footshock experience).

Supporting figure 4B depicts freezing displayed by ‘paired’ and ‘unpaired’ R_FE_ rats during the (S_FC_-R_FE_) pair-testing. The USV-footshock ‘paired’ rats froze significantly more than the USV-footshock ‘unpaired’ rats in reaction to USVs emitted by their partner S_FC_ rats (*P* < .01, by independent *t*-test).
